# The meaning of working in a person-centred way in nursing homes: a phenomenological-hermeneutical study

**DOI:** 10.1186/s12912-019-0372-9

**Published:** 2019-10-12

**Authors:** Tove K. Vassbø, Marit Kirkevold, David Edvardsson, Karin Sjögren, Qarin Lood, Ådel Bergland

**Affiliations:** 1 Department of Nursing, Faculty of Medicine, Institute of Health and Society, Oslo University, Oslo, Norway; 2 Lovisenberg Diaconal University College, Oslo, Norway; 3 Department of Nursing, Umeå University, Umeå, Sweden; 4 School of Nursing and Midwifery, La Trobe University, Melbourne, Australia; 5 Department of Health and Rehabilitation, Institute of Neuroscience and Physiology, University of Gothenburg, Gothenburg, Sweden

**Keywords:** Phenomenological-hermeneutical method, Person-centred care, Nursing homes, Thriving, Job satisfaction, Health care workers

## Abstract

**Background:**

The present study aims to illuminate the meaning of working in a person-centred way as experienced by staff in nursing homes. Insights into what working in a person-centred way mean for nursing home staff may contribute to a more comprehensive understanding of what gives staff satisfaction in their work and support further development of person-centred care approach in nursing homes.

**Methods:**

Interviews with 29 health care personnel who had participated in a one-year intervention focusing on person-centred care and thriving in three nursing homes in Australia, Norway and Sweden were performed, and a phenomenological-hermeneutical method was used to explore staffs’ lived experiences of working in a person-centred way in nursing homes.

**Results:**

For nursing home staff, working in a person-centred way meant that they were able to meet individual resident’s needs and expressed preferences in close family-like relationships, understanding the residents’ rhythms and preferences as the basis of the daily work plans and being able to do ‘the little extra’ for residents. Also, working in a person-centred way meant meeting shared goals by working towards a collective practice in collaborative teams. As a whole, the staffs’ lived experiences of working in a person-centred way in nursing homes was interpreted to mean thriving at work as a psychological state in which individuals experience both a sense of vitality and learning.

**Conclusions:**

Working in a person-centred way means staff thriving at work in nursing homes. The results further indicate that delivering care by only focusing on routines and practical tasks and not on residents’ preferences and well-being would inhibit thriving among nursing staff, leading to the potential for dissatisfaction with work.

## Background

The importance of having a sufficient number of competent nursing home staff to ensure quality of care is a major concern in many countries [1, 2]. High turnover and difficulties recruiting sufficient numbers of qualified staff are common challenges [3–5]. Several significant factors that may impact job satisfaction and the ability to provide quality care have been identified. These include unclear roles and functions [6], heavy workloads [7], demanding work schedules [8], difficult ethical issues [9, 10] and job strain, such as stress of conscience and burnout [11]. Dissatisfaction with work may result in lower loyalty to the workplace itself [12] and an increased probability of leaving the workplace [3]. Adverse health among staff may reinforce negative perceptions of nursing home work and further challenge recruitment and retention of competent staff [4, 13]. Furthermore, research indicates that those who are dissatisfied with their working conditions have an increased tendency to avoid work responsibilities through absence, purposely avoiding activities, taking shortcuts or making themselves unavailable when actions are required [14]. Therefore, there is a great need to identify ways to improve job satisfaction.

Person-centred care is increasingly being advocated as critical for good and effective dementia and nursing home care [15–17]. Influenced by a holistic view of man as a person, person-centred care emphasises patient-health care partnerships, recognising patient preferences and values, promoting care flexibility, and seeking to realise ethical paradigms of autonomy and right to self-determination [18, 19]. In nursing homes, person-centred care also requires developing relationships, promoting the residents’ well-being and contributing to meaningful lives for the residents [16, 20, 21]. Both climate and culture can describe the nature of a person-centred organisation. However, the concepts have been recognised as different. According to EH Schein [22], culture is invisible and about how group members think and believe, and can be expressed by using concepts such as beliefs, values, standards of behaviour and routines. Climate, on the other hand, means the physical arrangements and how a defined group of people interact with each other and can be described by the emotions it creates. Consequently, the overall experience of the organisation - the feelings arising from the physical and psychosocial environment and social interactions, can be described as the person-centred climate and represents a prerequisite for realising and also an integral part of person-centred care in nursing homes [20, 23].

Previous studies [24–27] have found associations between person-centred care and job satisfaction, and systematic reviews [28, 29] have indicated that person-centred care interventions may increase staff job satisfaction. Previous research [30] focusing on advantages of working in aged care, reported it as feelings of accomplishment and fulfilment as well as building compassionate relationships as essential values attached to working in aged care. However, the deeper understanding of the experiences and its meanings of staff working in a person-centred way in nursing homes settings have not been explored in depth.

This article reports from a sub-study of an intervention study aimed at promoting person-centred care and thriving in nursing homes [31]. The main intervention study was expected to provide evidence on how person-centred care may improve well-being and thriving among people who reside in nursing homes, and how person-centred care may improve staffs’ satisfaction with work. In this part of the study, we sought to explore in detail what working in a person-centred way means for nursing home staff by, in particular, focusing on their satisfaction from work.

## Method

### Aim

This study aims to illuminate the meaning of working in a person-centred way as experienced by nursing home staff, focusing on their satisfaction from work.

### Design

A phenomenological-hermeneutical design [32] was applied to open-ended individual interviews with nursing home staff narrating their experiences of working in a person-centred way.

### Setting

Three nursing homes that had participated in a previous intervention study [31] were included. One is located in a large city in Norway, and the other two are in rural areas in Australia and Sweden. The residents in all three nursing homes were diagnosed with dementia or with multiple chronic diseases. All facilities were publicly funded, and the number of beds ranged from 50 to 127.

### Recruitment and participants

Twenty-nine participants from the three nursing homes which had participated in the intervention were recruited by the local leaders in collaboration with the researchers according to the following inclusion criteria having worked at least 1 month in the nursing home during the intervention period and being willing to describe and talk about their experiences of working in the nursing home. Potential participants received a formal invitation, and all agreed to participate. All staff who confirmed a desire to participate were included. The participants gave their written consent to participate before the interviews were conducted. Twelve participants were recruited from one nursing home, while the two other nursing homes recruited nine and eight participants, respectively. The participants consisted of 10 registered nurses (RNs) (including three managers), 12 enrolled nurses (ENs), two care assistants (CAs), two personal carers, two occupational therapists and one physiotherapist. Twenty-seven of the participants were women, and three were men; the participants were 22 to 65 years of age with a range of 1 to 42 years of experience working in aged care (average of 12 years).

### Interviews

The research group included senior researchers with relevant experiences and methodological knowledge [31]. Data was collected through individual interviews, one with each participant. Open-ended questions were used to encourage thorough descriptions from staff regarding their perspectives on the meaning of working in a person-centred way (Additional file 1). To gain a deeper understanding and to clarify staff’s descriptions, the interviewees were asked to discuss their work as freely as possible and formulate their descriptions in their own words. They were asked questions such as, ‘what do you see as the most important when working in a person-centred way and what are your experiences?’, and ‘what do the experiences mean to you in your day-to-day work?’. The interviews lasted 30–60 min, were in the native language of each country and were tape-recorded and transcribed verbatim for analysis. In Australia the interviews were conducted by the senior researcher (DE) and the local project manager (QL). In Sweden they were conducted by the local project manager (KS), and in Norway, the interviews were conducted by the first author (TKV). All text was coded with a letter for site (A, N or S), and a number in the order of which the interviews were completed (e.g. A1, N1, S1). Field notes were not part of the data, and no software program was used to assist with searching or coding of the data [33]. The data was collected from September 2017 to March 2018, and the interviews took place in separate rooms in the nursing homes where participants could talk without being interrupted. As the researchers responsible for the intervention in the three countries also conducted the interviews, they were known to some of the participants.

### Analysis and interpretation

Data were analysed using Lindseth and Nordberg’s [32] phenomenological-hermeneutical method for researching lived experience. The method was inspired by Ricoeur’s [34] interpretation theory aimed at interpreting and understanding the meanings of a phenomenon, in this case, working in a person-centred way in nursing homes. The interpretation involves a dialectical movement between the text as a whole and parts of the text, and it moves through three interactive phases: naive reading, structural analyses and comprehensive understanding (critical interpretation) [34].

*Naive reading* was the first attempt to gain an overall impression of the text and its meaning and wholeness in light of the researchers’ pre-understanding of the topic. This gave access to the staff’s lived experiences with working in a person-centred way. The text was read several times, and the immediate impressions of what it meant for the staff to work in a person-centred way were written down. The objective of the second step, *structural analysis,* was to explain what is talked about in the text. In this phase, the text was separated into meaning units that were abstracted, coded and grouped, first as sub-themes and then as themes. The researchers went back to the original text several times to make sure that every sub-theme could be identified in the text, and conversely, that all relevant text was reflected in the themes. Themes and sub-themes are presented in the results section. The *comprehensive understanding* developed as the last step constitutes the discussion section. In this phase, the interpretation was guided by the researchers’ pre-understanding that working in a person-centred way is positive for experience of their work. The relationship between the residents’ positive experiences and states, and how staff experienced their work was prominent in the interviews. The themes were summarised and reflected on concerning the aim and the contexts of the study, taking into consideration the naive reading, the structural analysis, researchers’ pre-understandings and theory found relevant to what the interpretation opened for.

## Results

For staff, working in a person-centred way meant that they could meet individual resident’s needs and expressed preferences in close relationships and that they were meeting shared goals in collaborative teams. The results are presented in two themes and six sub-themes (See Table 1).

**Table 1:** Thematic structure of the meaning of working in a person-centred way

Themes	Sub-themes
Meeting individual resident's needs and preferences in close relationships	Being in close family-like relationships with residents Understanding residents’ rhythms and preferences as the basis of daily work plans Doing the ‘little extra’ for residents
Meeting shared goals in collaborative teams	Being part of a supportive team Working towards a collective practice Sharing professional values

### Meeting individual resident's needs and preferences in close relationships

#### Being in close family-like relationships with residents

Staff described that they enjoyed working in nursing homes because of the social relational aspects of work and the possibilities of getting to know the residents well through long-term relationships. For many, working with older people in a nursing home was a choice that reflected their values and beliefs:

‘Somebody else may say it’s just a job. To me, this is not just a job; it’s a fulfilment’ (A6).

The resident–staff relationships could become family-like friendships, where the residents and staff showed an interest in each other as persons and were described as sources for experiencing meaningfulness. Knowing the resident well made staff more aware of the resident’s wishes and preferences. Moreover, staff focus was not limited to the residents’ diagnoses and needs. It was equally important to create a comfortable and homely atmosphere to promote the residents’ well-being and thriving:

‘I’m not just there to help with their ADLs and get them prepared for the day or put them back to bed. There is more to it by creating a community or a family sort of feeling’ (A10).

Interactions with residents with cognitive impairment could be challenging for staff. For example, it could be demanding to interpret spoken words and subtle signs that initially seemed incomprehensible. When staff were able to understand the message and then meet these residents’ wishes, they experienced pride and satisfaction. One nurse described her sense of joy and enthusiasm from discovering successful solutions to meet the needs of residents with dementia:

Some days are quite exhilarating. You get to explore, − look below and ‘between the curtains’ so to say … Manage to find a way for her to fulfil her desire to go to the hairdresser and respect her at the same time; I enjoyed the feeling for a long time. I am smiling when thinking about it (N8).

#### Understanding residents’ rhythms and preferences as the basis of daily work plans

Staff emphasised the importance of following the residents’ routines and rhythms, such as when to get up, sleep, eat or socialise, for their daily working plans in order to promote well-being and thriving. By working in this way, they also experienced the necessary independence and authority to plan their work, make decisions and manage their responsibilities during the shift:

‘For that is how I want to work. I would not like to go here and only do some routine job’ (S4).

Confirming the individual resident’s rhythms positively affected the days for both residents and staff. The residents’ behaviours became more content and calmer, and staff experienced a better workflow.

#### Doing the ‘little extra’ for residents

Prominent in the text was the staff’s descriptions of how they deeply appreciated doing ‘the little extra’ for the residents. This could include going for a walk, reading a book, serving a glass of wine or a special cup of tea, sitting down for a chat, providing a massage, skincare or makeup, taking care of a resident’s flowers or playing scrabble or cards. Doing this little extra could create a calm and friendly atmosphere which spread through the environment and positively influenced persons other than those directly involved:

‘That is what makes me thrive; doing the little extra. If I do not have time to do the little extra, the job has no meaning to me’ (N5).

A positive comment, a smile, laughter, loving touch and hugs, or the fact that the resident seemed calm and relaxed were perceived as both a confirmation of proper care and as a reward. These experiences energised staff to perform other tasks and gave the work meaning. Furthermore, the staff used positive words such as fulfilment, enjoyment, satisfaction, well-being, thriving and pride to describe their experiences and feelings related to doing the little extra. These positive feelings of energy and fulfilment affected their home lives as well:

You have given her that moment … it’s really nice. You get to be so satisfied when you feel you have done a good thing, and she is happy … If you manage to be efficient in getting all the tasks done and also manage to do the little extra, you go home with a good conscience, and you are having a much more positive experience of the working day, and more energy when you go back home (N1).

### Meeting shared goals in collaborative teams

#### Being part of a supportive team

The participants emphasised strongly how vital the companionship with their colleagues was. The experiences of being seen, needed and supported by the team gave them a sense of satisfaction and meaning in their work:

Being part of a team, it’s really important to me. I really like working here because of that. It is quite crucial that we are honest with each other, besides are open and take care of each other (A9). Some days, there are no issues, and we achieve everything we need to without being stressed. On such days I think to myself; oh, how great this day has been! (S5).

The experience of being acknowledged, understood, supported and confirmed by the team could change a miserable and rather hard day into a feeling of being fortunate and happy at work:

‘It’s more pleasant to go to work when we recognise and see each other. Otherwise, I would not have continued to work here for as long as I have’ (S4).

Also, a high degree of flexibility in their work plans made it easier for staff to collaborate and consult with each other in short informal meetings and, further, to support and help each other during the shift:

I feel better when we work the way we do here. I experience less stress and pressure, even though the formal requirements are the same. It is positive for all, I think. We work all day here, and there is much more peace. It does something to the staff. I feel that the atmosphere it creates leads to that we have a satisfactory team relationship (N6).

Collegial support and understanding were particularly crucial in situations where a staff member felt embarrassed or inadequate in meeting the resident’s needs (e.g., having lost his/her patience in a particular situation when meeting repeated challenging interactions). A collegial, supportive team allowed for the necessary openness to ask for advice to deal with uncomfortable situations. Often the team collaborated to find an optimal solution together.

#### Working towards a collective practice

Performing care from a common everyday basis and towards agreed upon goals for practice provided a stable foundation for their work. Having a common understanding enabled staff to put differences or disagreements aside and work together to produce positive outcomes by planning and evaluating the care plans across different professions. Additionally, a shared understanding and concern for the residents made staff perceive positive and useful outcomes by consulting each other and discussing the best way to do work:

‘All the nurses do the same kind of work here, and I think this promotes well-being and thriving in the staff group because it makes us feel equal’ (N6).

By sharing with colleagues how they performed care to meet residents’ needs, individuals learned from each other and achieved mutual respect for individuals’ skills. Hence, the collective agreement resulted in strengthening the collective knowledge regarding caring for persons with dementia:

We cooperate with the care tasks to perform care in the best possible way. So, if I fail, maybe someone else can try. ‘Playing’ on each other’s talents and ask the ones who have succeeded, what and how did you do, that is often just what is needed (N6).

To trust in, respect and depend on each other’s competence made it less challenging to put aside or cross professional boundaries. The collective understanding of how to perform and realize care, promotes effective communications, the role of recognition and valuing each other’s contribution to reach their goal of providing the best life possible for the residents:

‘People who are thinking the same way allow you to do the work and to be natural with what you do’ (A7).

#### Sharing professional values

Staff emphasised the importance of working in a team in which colleagues had common values underlying their work for them to feel satisfied and to thrive:

You must bring your heart to work and be willing to and wish to work with frail residents. You must be aware of the standards on which the work is grounded. You are here because you want to be and because you want to perform good care (N2).

Also, shared values were regarded as vital to further developing professional values and integrity, refining their skills, supporting further learning and development of their practice:

I have experienced that colleagues that are sharing the goals and want to work in this field increase their professional competence and are more likely to wish to learn more, such as about environmental measures to promote person-centred care for instance (N2).

## Comprehensive understanding and discussion

The results highlighted that working in a person-centred way could be conceptualized in terms of two themes, namely: Meeting individual resident's needs and preferences in close relationships, and Meeting shared goals in collaborative teams (themes) and the subthemes Being in close family-like relationships with residents, Understanding residents’ rhythms and preferences as a basis for daily work plans, Doing the ‘little extra’ for residents (theme one), Being a part of a supportive team, Working towards a collective practice and Sharing professional values (theme two).

To arrive at a comprehensive understanding of the meaning of working in a person-centred way, the thematic structure and associated experiential descriptions from staff were interpreted in light of the theoretical work of G Spreitzer, K Sutcliffe, J Dutton, S Sonenshein and AM Grant [35]. They describe thriving at work as a psychological state in which individuals experience both a sense of vitality and learning. Thriving is thus in their description about personal growth and development [36]. Vitality is described as the positive experience of having energy, learning refers to acquiring and seeking new knowledge and skills. Similarly, our results indicate that the opportunity to work in a person-centred way was experienced to give staff a sense of vitality by providing the energy to meet residents’ needs in a way that acknowledged their preferences. Despite describing the work as hard and demanding at times, gratifying responses from the residents was experienced to give staff energy and inspiration to continue doing their job. Moreover, staff also described what they learned from their own experiences and those of their colleagues provided inspiration and energy to continue to deliver person-centred care.

Based on the thriving at work model by Spritzer et al. [35], the descriptions of working in a person-centred way can be interpreted as agentic work behaviours, which encompass task focus, exploration and heedful relating. Task focus describes the moments when an individual is attentive and alert during the performance of the assigned work-related tasks [37]. In nursing, ‘task focus’ could be perceived as ‘care focus’, understood as focusing on meeting and fulfilling the assigned responsibility caring for others (as opposed to carrying out administrative duties or housekeeping tasks). Hence, ‘task focus’ concerning thriving at work is a different conception compared to the task focus that person-centred care models are aimed at replacing; focusing on completing tasks rather than caring for persons [15]. Exploration is described as innovation behaviours that help people to stretch and grow, and heedful relating is explained as acting intentionally and being self-directed, active and purposeful. When staff described working in a person-centred way, they elaborated how they were focusing on meeting each resident’s individual needs, adjusting to each residents’ rhythm and improving residents’ well-being by doing the little extra (care focus). Recognising a need for improvement was described by staff as motivating them to explore and identify new ways to approach the residents, and to ask for assistance or guidance from colleagues to accomplish success in their work (exploration). Thus, the staff’s descriptions indicate that focusing on what they perceived as their primary care actions is an essential and integral part of the learning process.

While doing the little extra primarily seemed to be an individual performance, much of the work described was completed as part of a team. When they worked together in a group that shared the same purpose, namely to provide person-centred care, staff described how they became aware of how their contributions were connected. Moreover, when helping each other to accomplish the work, they were attentive towards colleagues who needed assistance and provided support for them (heedful relating [35]). Acting heedfully for each other involved that staff felt comfortable and safe about asking colleagues for assistance or advice. Due to the careful interactions between the colleagues, learning arose as staff refined their skills and gained new knowledge. Additionally, the heedful interactions promoted vitality and further motivation for work. Thus, the results can be interpreted through the lens of Spritzer et al. [35], as working on a person-centred way meant that staff were carefully related to each other, which in turn were expressed of staff as developing feelings of vitality and learning and a continued effort to explore, learn and grow on the job.

Moreover, when working in a person-centred way (i.e., carrying out agentic work behaviours), resources were produced [35]. Knowledge resources were created from collegial discussions of new ways of solving difficult or challenging situations, in which they shared experiences and learned from each other. Thus, task-focusing (care focus), exploration and heedful work behaviours could be interpreted as being involved in the process of knowledge creation. Being in close relationships with residents as well as colleagues could be interpreted as resulting in relational resources as well as positive feelings. In sum, our findings seem to illustrate the thriving of work model of Spreitzer et al. [35] that an interaction exists between agentic work behaviours and the resources that lead to thriving. These feelings encouraged them to continue heedfully relating with each other, which in turn nurtured agentic work behaviours and sustained thriving. Our findings can exemplify the thriving at work model because the interviewees experienced positive feelings like fulfilment, meaning and joy from interactions with residents and colleagues, which gave them a sense of vitality and learning. These feelings encouraged them to continue heedfully relating with each other, which in turn nurtured agentic work behaviours and sustained thriving.

Furthermore, according to Spreitzer et al. [35], the propensity for agentic work behaviours can be heightened and stimulated by three contextual features: decision-making discretion, broad information sharing and a climate of trust and respect. When considering our results, we see that the participants emphasised the importance of being allowed to make decisions for meeting individual residents’ needs and expressed preferences as well as being able to do the little extra for residents (decision-making discretion). Furthermore, working in a person-centred way meant that staff had access to information which they shared with their teams, contributing to their ability to confront challenges and providing staff with a sense of how their work fits with others and the overall shared goals (broad information sharing). Moreover, their descriptions demonstrated that working in a person-centred way meant being self-governing and autonomous at work; this result may elucidate that staff were valued and respected in their organisations as individuals and professionals (a climate of trust and respect). Thus, it can also be interpreted that the processes of working in a person-centred way that leads to thriving are connected with the qualities created in the working environment.

Previous research focusing on which qualities of work foster job satisfaction have increasingly emphasised that well-being and satisfaction come from individual achievements and personal growth at work [38, 39]. This was also supported in a recent study of nursing home staff [40], which found that a healthy working environment in aged care depended on if staff were empowered to use their creativity and knowledge when performing that care. The current study indicates that the nursing home context has the potential for developing professional and personal growth, leading to thriving and satisfied staff. Another recent study [30] found that the advantages experienced resulted from the relationships with the residents and affirmative teams as well as autonomy in daily tasks. Thus, based on our interpretation, we suggest that adapting person-centredness to the care culture in nursing homes can be regarded as an organisational intervention for making the work more challenging and motivating, which will promote vitality and learning, and hence, staff will thrive at work.

### Strengths and limitations

A strength of this study is that the employees in three different nursing homes in three different countries provided rich descriptions of their experiences of working in a person-centred way concerning their experiences of satisfaction with work. The commonalities in experiences across variation in context increase the understanding of the relationship between person-centred care and the staffs’ work experiences. Despite efforts to include both genders, the participants consisted mainly of female participants. The staff mix may have an impact on the transferability in general and male care workers in particular, although the gender of the participants reflects the entire staff at the included nursing homes.

There could be limitations of the interviews and analysis due to the researchers being sensitive to the concepts of the intervention study [31]. To ensure the trustworthiness, the method for researching lived experience of Lindseth and Norberg [32], inspired by Ricoeur [34], was used throughout the research process. The broad knowledge and experiences of the research team in terms of person-centred aged care and qualitative research contributed to credibility and dependability. According to Ricoeur, the preunderstanding can never be disregarded but should be made explicit; this was achieved by reflecting and discussing the preunderstanding of the members of the research team throughout the research process.

## Conclusion

For nursing home staff, working in a person-centred way meant that they could meet individual resident’s needs and expressed preferences in close relationships meeting shared goals in collaborative teams. By interpreting these results in light of Spreitzer et al.’s [35], model of thriving at work, the understanding of the processes contributing to thriving among staff is further deepened. Our results underscore the importance of reflecting on how to develop working environments that promote thriving at work and that Spreitzer’s model may be useful in identifying important factors that may contribute to succeed in reaching this goal.

## Supplementary information

**Additional file 1.** Interview guide.

## Data Availability

Not applicable. The data will not be shared. Ethics approval for the study requires that the transcriptions of the interviews are kept in locked files, accessible only by the authors.

## Abbreviations

ADL: Activity of Daily Living
CA: Care Assistant
EN: Enrolled Nurses
RN: Registered Nurses
