# Supplementary material for: The meaning of working in a person-centred way in nursing homes: a phenomenological-hermeneutical study
Source: BMC Nurs. 2019 Oct 12;18:45. doi: 10.1186/s12912-019-0372-9 (PMC6790040; doi:10.1186/s12912-019-0372-9)
Supplement: Supplementary file 1 — Additional file 1. Interview guide. [file 12912_2019_372_MOESM1_ESM.docx]

**Interview guide for individual interviews with staff that participated in:**

*‘A person-centred and thriving-promoting intervention in nursing homes - study protocol for the U-Age nursing home multi-centre, non-equivalent controlled group before-after trial’ (Edvardsson et al., 2017).*

Questions that guided the interviews:

1. If you think back, can you please tell me something about a situation or meeting that you experienced as person-centred?
   1. What do you think made this situation person-centred?
   2. What did this situation mean to you?
2. What does working in a person-centred way mean for:
   1. you as a nurse/healthcare worker?
   2. the residents?
   3. the next of kin?
   4. your colleagues and the staff group as a whole?
3. Do you have any additional questions/comments/suggestions?

Reference:

Edvardsson, D., Sjögren, K., Lood, Q., Bergland, Å., Kirkevold, M., & Sandman, P.-O. (2017). A person-centred and thriving-promoting intervention in nursing homes-study protocol for the U-Age nursing home multi-centre, non-equivalent controlled group before-after trial. *BMC Geriatrics, 17*(1), 22.
